# Heterogeneity among *Mycobacterium ulcerans* from French Guiana Revealed by Multilocus Variable Number Tandem Repeat Analysis (MLVA)

**DOI:** 10.1371/journal.pone.0118597

**Published:** 2015-02-23

**Authors:** Yann Reynaud, Julie Millet, David Couvin, Nalin Rastogi, Christopher Brown, Pierre Couppié, Eric Legrand

**Affiliations:** 1 Institut Pasteur de la Guyane, Cayenne, French Guiana; 2 Institut Pasteur de la Guadeloupe, Pointe à Pitre, Guadeloupe; 3 National Oceanic and Atmospheric Administration, Milford, Connecticut, United States of America; 4 Centre Hospitalier André Rosemon, Cayenne, French Guiana; University of Padova, Medical School, ITALY

## Abstract

Buruli ulcer is an emerging and neglected tropical disease caused by *Mycobacterium ulcerans*. Few cases have been reported so far in the Americas. With 250 cases reported since 1969, French Guiana is the only Buruli ulcer endemic area in the continent. Thus far, no genetic diversity studies of strains of *M. ulcerans* from French Guiana have been reported. Our goal in the present study was to examine the genetic diversity of *M. ulcerans* strains in this region by using the Multilocus Variable Number Tandem Repeat Analysis (MLVA) approach. A total of 23 DNA samples were purified from ulcer biopsies or derived from pure cultures. MVLA was used in the study of six previously-described Variable Number of Tandem Repeat (VNTR) markers. A total of three allelic combinations were characterized in our study: genotype I which has been described previously, genotype III which is very similar to genotype I, and genotype II which has distinctly different characteristics in comparison with the other two genotypes. This high degree of genetic diversity appears to be uncommon for *M. ulcerans*. Further research based on complete genome sequencing of strains belonging to genotypes I and II is in progress and should lead soon to a better understanding of genetic specificities of *M. ulcerans* strains from French Guiana.

## Introduction

*Mycobacterium ulcerans* is a slow-growing tropical and subtropical bacterial pathogen that causes skin ulcerations. Buruli ulcers (BU) are symptomatic of *M*. *ulcerans* infection, which is classified by the World Health Organization (WHO) as one of 17 neglected tropical diseases. It has been reported in at least 34 countries and is endemic to some parts of Southeast Asia, Australia and is more prominent in West and Central Africa where it is recognized as a serious health problem. Few cases have been reported so far in South America, thus far only in Peru, Mexico, Suriname and French Guiana (FG) [1]. However, as a consequence of the nearly 250 cases reported in FG since 1969 (average incidence of 2.09/100,000) [2], FG qualifies as the only BU endemic area in the Americas. This could be explained by the efficacy of BU diagnosis in FG, or may possibly be a consequence of genetically distinct *M*. *ulcerans* pathogens, as compared with strains found in other countries in Americas. This hypothesis was addressed in the present study.

Several genotyping methods have been applied to *M*. *ulcerans*, but because of restricted genetic diversity of this “monomorphic” species, multilocus variable number tandem repeat analysis (MLVA) remains the preferred approach for epidemiological studies of BU [3–8]. Only two strains (ITM7922 and Mu_1G897) from FG have been genotyped previously [4,9]; we therefore used MVLA to examine the genetic diversity of clinical *M*. *ulcerans* strains in this region. We report herein the first evidence of a high degree of genetic heterogeneity among strains of *M*. *ulcerans* isolated in FG.

## Materials and Methods

Genetic diversity of *M*. *ulcerans* samples isolated in FG between 1994 and 2013 was assessed by sequencing of VNTR (Variable Number of Tandem Repeats) loci. A total of 23 ulcerated BU tissue biopsies were first decontaminated with BB MycoPrep Specimen Digestion/Decontamination Kit, then cultured in Löwenstein-Jensen medium (BD Biosciences). Only 6 positive cultures were obtained after 6 weeks for strains MuFG11, 100, 102, 123, 124, 125 (Table 1). DNA samples were purified by QIAamp DNA Mini Kit (Qiagen) either from positive bacterial cultures (6/23) or directly from decontaminated biopsies when cultures were negative (17/23). DNA samples were extracted separately from 1994 to 2013 for diagnostic purposes. All purified DNA samples were tested by PCR for the presence of IS2606 [10] and ketoreductase B (KR) domain of the mycolactone polyketide synthase (mls) gene from the plasmid pMUM [11]. Presence of IS2404 was tested by Taqman (Life Technologies) real time PCR and amplicons were detected on a 7300 real-time PCR System (Applied Biosystems) using ULC5 (GTCGCCGAGAAAAGCAATGA) and ULC6 (GACTTCAAGGTGGCGCAGAT) primers and ULCS01 (FAM-ATGCGATGCATACCCA-MGBNFQ) probes following this protocol: 1 cycle of 50°C for 2 min, 1 cycle of 95°C for 15 min, 40 cycles of 95°C for 15 s and 60°C for 1 min. Molecular typing was performed using six previously-described VNTR markers: VNTR18 and VNTR19 [3], mycobacterial interspersed repetitive units (MIRU) MIRU5 and MIRU33 [7], ST1 [6] and microsatellite SSR [5]. For unamplified VNTR PCR products or weak amplifications (Table 1), six nested PCR assays were developed using specifically designed primers (Table 2), with the annealing temperature set at 58°C. Negative controls were included for diagnosis and VNTR typing steps, consisting of PCR amplifications without DNA. The strain MuFG100 obtained from pure culture and identified by Whole Genome sequencing (WGS) as a “classical” FG genotype I *M*. *ulcerans* strain (data not shown), was used as a positive control in our study. All PCR procedures were carried out on a Mastercycler Gradient (Eppendorf) using HotStar Taq DNA polymerase (Qiagen), following the manufacturer’s instructions. Sequencing of VNTR PCR products was carried out by Beckman Coulter Genomics (United Kingdom) using an ABI PRISM 3130xl Genetic Analyzer (Applied Biosystems). Sequences were aligned using CLUSTAL W [12].

**Table 1:** Characteristics of *M*. *ulcerans* samples used in this study and sequence types based on sequence variations of tandem repeat units.

DNA	Geographic origin	Month/year of sampling	Age of patient	VNTR18	VNTR19	MIRU5	MIRU33	ST1	SSR No. of repeat	Genotype
MuFG1	FG	08/2008	37	**J**	**LDK**	**A**	A	**C**	10	I
MuFG7	FG	03/2009	16	**J**	**LDK**	**A**	**A**	**C**	**10**	I
MuFG11*	FG	10/2010	56	**J**	**LDK**	**A**	**A**	**C**	**10**	I
MuFG13	FG	06/2008	79	**KLM**	**MNDK**	**A**	**AA**	**GD**	**26**	II
MuFG15	FG	06/2008	66	**J**	**LDK**	**A**	**A**	**C**	**10**	I
MuFG18	FG	08/2004	33	**J**	**LDK**	A	A	**C**	10	I
MuFG22	FG	10/2004	18	**J**	**LDK**	**A**	A	**C**	10	I
MuFG25	FG	10/2004	52	**J**	**LDK**	**A**	**A**	**C**	**10**	I
MuFG26	FG	08/2004	14	**J**	**LDK**	**A**	**A**	**C**	**10**	I
MuFG36	FG	01/2001	40	**J**	**LDK**	**AB**	**A**	**C**	10	III
MuFG58	FG	04/2002	38	**KLM**	**MNDK**	**A**	**AA**	**GD**	**26**	II
MuFG64	FG	07/2002	50	**J**	**LDK**	**A**	A	**C**	**10**	I
MuFG68	FG	08/2002	33	**J**	**LDK**	A	A	**C**	10	I
MuFG86	FG	09/2003	66	**J**	**LDK**	**A**	**A**	**C**	**10**	I
MuFG95	FG	05/2012	43	**J**	**LDK**	A	**A**	**C**	**10**	I
MuFG96	FG	06/2004	36	**J**	**LDK**	A	**A**	**C**	**10**	I
MuFG100*	FG	04/2011	49	**J**	**LDK**	**A**	**A**	**C**	**10**	I
MuFG102*	FG	02/2013	37	**KLM**	**MNDK**	**A**	**AA**	**GD**	26	II
MuFG117	FG	07/2013	57	**J**	**LDK**	A	A	**C**	10	I
MuFG118	FG	08/2013	36	**KLM**	**MNDK**	A	AA	**GD**	26	II
MuFG123*	FG	05/1994	12	**J**	**LDK**	**A**	**A**	**C**	**10**	I
MuFG124*	FG	09/1994	33	**J**	**LDK**	**A**	**A**	**C**	**10**	I
MuFG125*	FG	05/1995	37	**J**	**LDK**	**A**	**A**	**C**	**10**	I
Agy99	Ghana	1999	-	B	FGH	A	ABA	BD	34	African
128FXT	USA *X*. *tropicalis*	2001	-	J	K	AB	AA	GD	36	-

VNTR, Variable Number Tandem Repeat; MIRU, Mycobacterial Interspersed Repetitive Unit; FG, French Guiana;

* DNA purified from culture; in bold results obtained after PCR and sequencing without needing nested PCR

**Table 2:** Primer sequence for each VNTR marker amplified by nested PCR.

Marker	Forward primer	Reverse primer
VNTR18	GTACGTTGCGGGAACCTCT	GGAGCCTACGAATCTCATCG
VNTR19	GGCCATCAAGACATGGAGTT	ACGGGAGCGACTTCACAC
MIRU5	CGACAGCGATCAACCTGAC	CTGACTTCGGAATCGTCGTC
MIRU33	CGCACGCAGAAAATCTGG	ATCGAGTTGTCGGACACGA
ST1	GAGCAGGGGCTGGTGAAC	GACGACGAGGCGGTAGTG
SSR	ATTGATCACGGTGGGTATCG	GTCGGTAACCAAGACGGTGA

**Fig 1:**
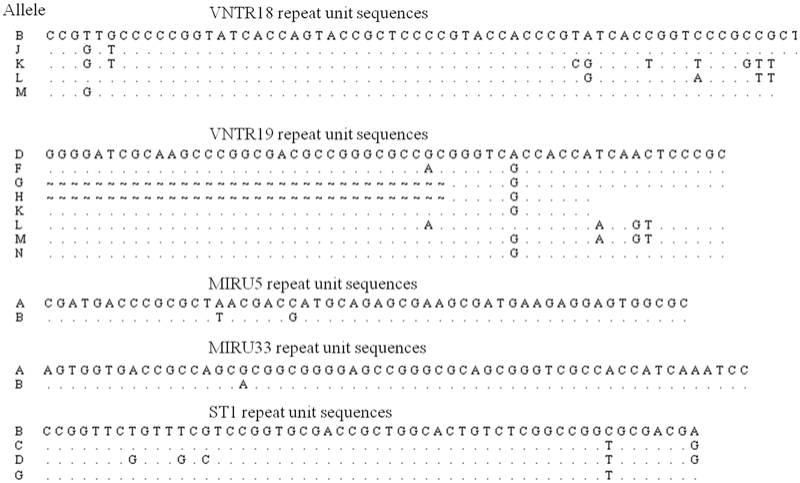
Sequence variation of tandem repeat units. Dashes indicate a base deletion, and dots indicate an identical base compared to the reference sequence. Letters on the left indicate allele sequence codes.

The complete sequences of two other strains were used in this study: the clinical strains *M*. *ulcerans* Agy99 [13] and *M*. *ulcerans* ecovar *liflandii* 128FXT isolated from the frog *Xenopus tropicalis* [14]. Repeated units were identified using Tandem Repeat Finder [15] and a sequence type was assigned for each strain depending on VNTR marker sequences (Table 1 and Fig. 1) and following sequence profiles previously described for VNTR18, VNTR19 [4] and ST1 [6]. Finally a genotype was assigned for each isolate. VNTR profiles of various well-studied strains of *M*. *ulcerans* [3,5,7,8,16] were added in the UPGMA analysis: a tree was built for all strains based on amplicons size for VNTR18 and VNTR19, and on the number of tandem repeats for MIRU5, MIRU33 and SSR markers in order to maximize the number of strains for comparison purposes. A Minimum Spanning Tree (MST) was built based on FG isolates and on whole genome sequences of Agy99 and 128FXT using sequence types assigned for all markers and using MLVA Compare V1.03 software (Genoscreen; Lille, France).

Analyzed samples were all obtained by tissue biopsy collections required by the standard medical diagnosis and care for any patient presenting BU symptoms on hospital admission in FG. Only one author (head of the dermatology unit at the Cayenne hospital) was involved in obtaining the tissue samples from the patients as part of the BU diagnosis. No identifying information was collected with the tissue samples for research studies. According to the French legislation (article L.1211–2 as related to the French Public Health Code), biobanking and secondary use for scientific purpose of human clinical remaining samples are possible as long as the corresponding patients are informed and they have not raised any objection to them. French legislation is fully accepted in FG. In the present research, this requirement was fulfilled: information was provided to every patient through the Hospital brochure entitled “Information for patients”, and no immediate or delayed patient opposition was reported by the hospital clinicians. No institutional review board approval is required according to the French legislation. Moreover, in keeping with French legislation (article L.1243–3 and related of the French Public Health Code), samples received were registered for research purposes in a biobank declared to both the French Ministry for Research and a French Ethics Committee (biobank name DC-2010–1223; collection N°2 Laboratoire de parasitologie/CNR chimiorésistances, deposited at the Institut Pasteur de la Guyane in FG). Biobanking was approved by Cayenne hospital. The database was declared to the Commission Nationale Informatique et Libertés (CNIL number 3x#02254258).

## Results and Discussion

**Fig 2:**
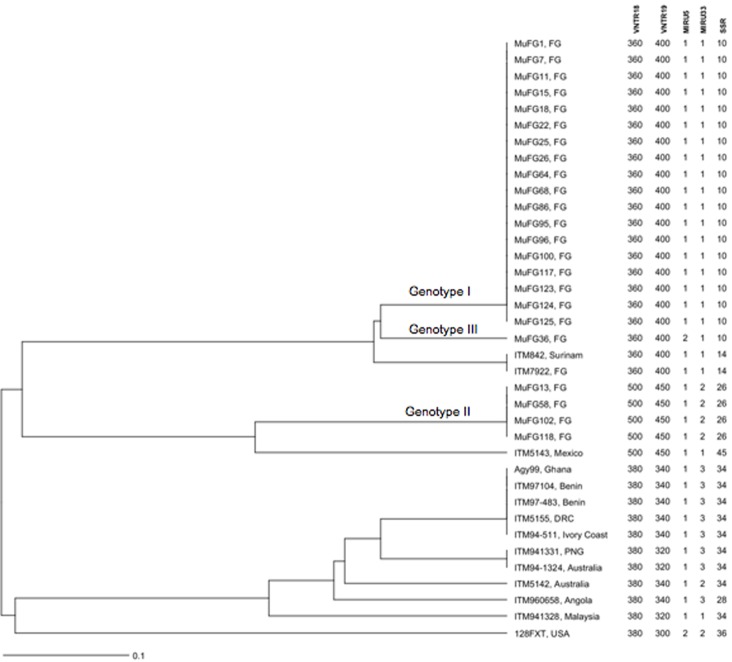
UPGMA phylogenetic tree. The analysis is based on amplicons size of VNTR18 and VNTR19 makers, and tandem repeats number for MIRU5, MIRU33 and SSR; FG, French Guiana; DRC, Democratic Republic of Congo; PNG, Papua New Guinea.

**Fig 3:**
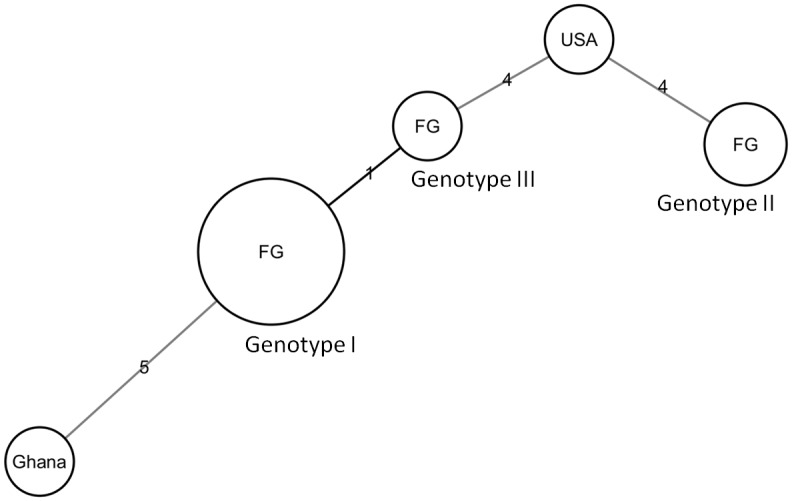
Minimum Spanning Tree. The analysis is based on sequence type for 6 VNTR markers where each circle is a graphical representation of sample size of each genotype (although not strictly proportional); the number of alleles differing between genotypes is indicated on each branch; FG, French Guiana.

All studied *M*. *ulcerans* DNA samples from FG were positive for IS2404, IS2606 and KR. A total of 84.8% (117/138) of VNTR markers were amplified directly by PCR and fully sequenced (Table 1). For unamplified VNTR markers remaining and for some amplifications that were too weak for sequencing purposes, nested PCR were carried out. A total of three allelic combinations were characterized in our study of those strains (Table 1, Fig. 2 and Fig. 3). A majority of these strains (18/23) belonged to genotype I, close to the “French Guiana” genotype reported in the well-studied strain ITM7922 [4,7,8]; the only difference found consisted of 10 repetitions of the SSR marker in our study, as compared to 14 reported in an earlier analysis [5]. Genotype III was represented only by MuFG36. However, this pattern bore a strong resemblance to genotype I with differences restricted to a single marker MIRU5, as well as to the strain ITM842 isolated in Suriname (Fig. 2). Sequence data from the final allelic combination, genotype II, revealed unique characteristics. A total of 4 of the 23 tested isolates from FG (MuFG102 from pure culture) belonged to this genotype with differences highlighted for each VNTR marker except for MIRU5. Genotype II is characterized by a total of 12 repetitions (when excluding SSR marker) as opposed to 7 for genotype I. New patterns of sequence variations (VNTR18 K, L, M, VNTR19 M, N and ST1 G) were identified in this previously undescribed genotype. Furthermore, the SSR profile (26 repetitions) has not been reported earlier (Fig. 1). Some markers, e.g., VNTR18, VNTR19 and ST1, appeared to be more polymorphic than others such as MIRU5 and MIRU33. Genotype II is closer to the strain ITM5143 isolated in Mexico (Fig. 2) than to the other genotypes found in FG. It noteworthy that genotype II bears more similarity to strain 128FXT than to other genotypes based on VNTR marker sequences (Fig. 3). The strain 128FXT was first considered as a mycolactone-producing *Mycobacteria* (MPM) named *M*. *liflandii* and responsible for *M*. *ulcerans-*like disease in a colony of the African frog *X*. *tropicalis* at the University of California, Berkeley (USA) in 2001 [17]. After WGS this strain was reclassified as *M*. *ulcerans* ecovar *liflandii* [14]. Other MPM (*M*. *shotsii*, *M*. *pseudoshotsii*, some *M*. *marinum* strains) have been described previously, and in association with *M*. *ulcerans*-like disease in ectotherms (fishes and frogs). Those strains appear genetically close to FG and Suriname strains by MLVA [16,18]. Furthermore, a phylogenomic analysis based on genome-wide SNPs [9] showed that those strains cluster together within “lineage 1”, while other *M*. *ulcerans* strains from Africa and Australia belong to “lineage 3”. Lineage 1 may then constitute an intermediate stage in the reductive evolutionary process between the *M*. *marinum* progenitor and other lineage 3 *M*. *ulcerans* strains. Indeed, Lineage 1 strains contain some versions of the *M*. *ulcerans* plasmid pMUM [19,20], the number of pseudogenes and deleted genes is substantially less important and the IS2606 copy numbers, chromosomal rearrangements and DNA deletions are smaller than in other *M*. *ulcerans* strains commonly involved in human infections, suggesting that they may have adapted to slightly different environmental niches as a consequence of their greater pleomorphic capacities [9,14,18,21].

The high degree of genetic diversity among strains from FG appears uncommon for *M*. *ulcerans*, which has been characterized by a very low level of genetic variability and characterized as “monomorphic”. Few MLVA studies have succeeded so far in the description of *M*. *ulcerans* genetic variability within a country: e.g., “Victoria” and “Southeast Asia” genotypes have been characterized using MIRU markers in Australia, although samples were localized at very distant locations [7]; “Southeast Asia” and “PNG genotypes” in Papua New Guinea (PNG) [7]. Four genotypes in the Democratic Republic of Congo were isolated over a large geographic distance [8]. A single study reported significant polymorphism within a restricted area in Ghana, in which 3 allelic combinations of ST1 and MIRU1 markers were identified [6].

**Fig 4:**
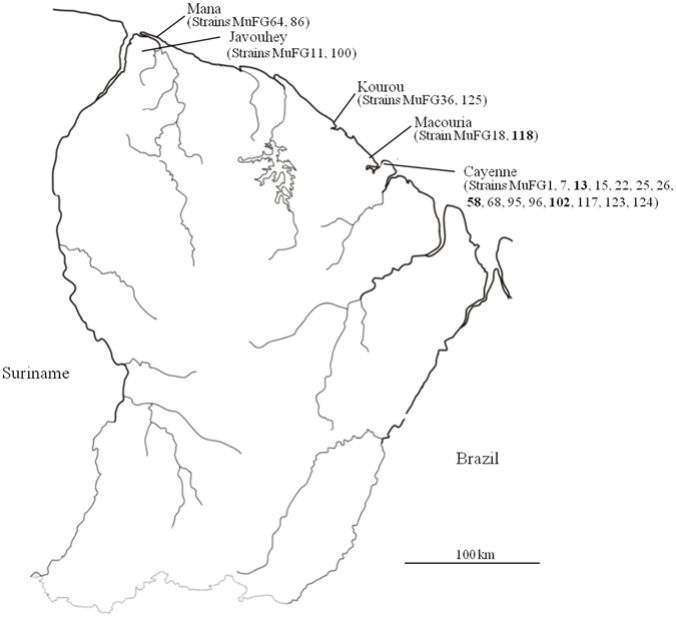
Localization of *M*. *ulcerans* samples in French Guiana. In bold isolates belonging to genotype II.

Finally, in our study, we did not observe any obvious correlations between the clustering of strains obtained by MLVA and the age of patients, date of infection or geographic localization (Table 1). All genotypes were localized along the FG coastline (Fig. 4) where most of the human population resides.

Further research is in progress based on WGS of MuFG11, 100 and 102 strains belonging to genotype I and II isolated by culture. The objective is to improve our understanding of the genetic diversity of *M*. *ulcerans* strains from FG. SNPs potentially identified by WGS will then be screened on biopsy samples in FG to validate their relevance for epidemiological survey. Such high throughput sequencing as performed previously in Ghana led to SNPs identification in *M*. *ulcerans* [22] increasing the resolving power of genotyping. Furthermore WGS should help to evaluate the reductive evolution processes involved and the position of *M*. *ulcerans* strains from FG (and more specifically genotype II) in these processes between the *M*. *marinum* progenitor, MPM and other *M*. *ulcerans* strains. For this we will focus on IS, pMUM, pseudogenes, chromosomal rearrangements DNA deletion, and *M*. *ulcerans* regions of divergence (MURDs). It is interesting to note that *M*. *ulcerans* strains from FG are the only strains pathogenic for humans (not for ectotherms) and belonging to lineage 1 [9]. Moreover the greatest IS2404 expansion (close to 300 copies) is found in the strains Mu_1G987 from FG, compared to around 200 copies in other *M*. *ulcerans* [9]. All of these characteristics in FG strains are worthy of more deep exploration in terms of their genomic specificities. Finally, WGS should help to explore environmental adaptation capacities by the examination of CDSs and pseudogenes and more specifically those associated with lipid metabolism, cell wall and cell processes.

## Conclusion

This study demonstrates for the first time genetic heterogeneity among *M*. *ulcerans* from FG with identification of three distinct genotypes. These results suggest that MLVA is a relevant first step approach for *M*. *ulcerans* genotyping in this area. A recent study [2] allowed the identification of *M*. *ulcerans* DNA from water samples in different parts of FG, but the link between clinical isolates and environmental samples has not been explored. Further research is in progress to characterize such link and to explore genetic diversity of environmental *M*. *ulcerans* DNAs.
